# The Effect of Florfenicol Given by Nebulization in the Treatment of Naturally Infected Calves With Bovine Respiratory Disease Complex: Randomized Clinical Study

**DOI:** 10.1002/vms3.70238

**Published:** 2025-02-07

**Authors:** Umit Ozcan, Mehmet Tutuncu

**Affiliations:** ^1^ Department of Veterinary Internal Medicine, Faculty of Veterinary Medicine Ondokuz Mayis University Samsun Türkiye

**Keywords:** bovine respiratory disease, calf, florfenicol, inhalation therapy, nebulization

## Abstract

**Background:**

Bovine respiratory disease (BRD) is still one of the major problems for herd management due to the negative effects on herd health, costs due to treatment, reduced weight gain and calf loss. The aim of this study is to evaluate the effectiveness of aerosolized florfenicol in calves with naturally infected BRD.

**Methods:**

Forty‐five calves were included in the study and divided into three groups. Group 1 received florfenicol subcutaneous route. While Group 2 received florfenicol administered solely through nebulization, Group 3 received florfenicol via nebulization in addition to flunixin meglumine administered intramuscularly (IM). BRD pathogens were determined from bronchoalveolar lavage (BAL) samples. The treatment period was monitored with a clinical respiratory score, haematology, thorax ultrasonography and serum haptoglobin levels.

**Results:**

*Mycoplasma bovis* was the main primary bacterial pathogen isolated from BAL fluid, *Escherichia coli* was the main secondary bacterial pathogen and bovine respiratory syncytial virus (BRSV) was found to be the primary viral BRD pathogen. The treatment period was shortened to the 2nd day in the groups with nebulization. Calves with clinical respiratory scores of 12 and above died in all groups. There was no significant difference in lung ultrasonographic scoring and haematology results before and after treatment within the groups. There was a significant decrease in the high haptoglobin values before and after the 3rd day of treatment. The study's main limitation was that there was no negative control group in this study due to ethical reasons.

**Conclusion:**

It was concluded that florfenicol administered by inhalation in BRD patients is more effective, reduces the recovery time and will be a promising treatment strategy.

## Graphical abstract

*M. bovis* and BRSV were the main pathogens in BRD calves. No PI isolated in any animals. Haematology, haptoglobin levels and thoracic ultrasonography scores were not significant. Reduction in clinical respiratory score was better in nebulized therapy groups after the second day.

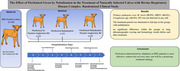

## 1 Introduction

The term ‘bovine respiratory disease’ refers to an undifferentiated disease of cattle characterized by dyspnoea, coughing, nasal discharge, pneumonia in clinical examination and non‐specific signs of toxaemia. The BRD is caused by numerous combinations of infectious agents, host defences and environmental conditions (Ollivett and Buczinski 2016).

The primary pathogens of BRD are ubiquitous, and all the primary bacterial respiratory pathogens are commensal in clinically normal cattle. It is proposed that clinical BRD is the result of the effect of various stressors causing immunosuppression, thereby allowing the colonization of the lower respiratory tract by opportunistic pathogens that are invariably encountered by cattle (Bassel et al. 2020).

In animals in which the immune system is weakened by stress, viral infections such as bovine herpes virus 1 (BHV‐1), parainfluenza 3 (PI3), bovine viral diarrhoea virus (BVDV) and BRSV may become established, leading to a breakdown of the normal defence mechanisms of the upper respiratory tract without causing any disease and are then capable of invading lung tissue and causing pneumonia and lung damage. It is a combination of stress and exposure to viruses and bacteria that leads to the development of BRD. It is already known that whilst *Mannheimia haemolytica* and *Pasteurella multocida* are common causes of bacterial BRD, several other species of bacteria (*Mycoplasma bovis*, *Mycoplasma* spp., *Escherichia coli* and *Trueperella pyogenes*) can fill this niche if the opportunity arises (Catania et al. 2020; Portis et al. 2012).

Since BRD is caused by bacterial, viral and mycoplasmal agents, the first step recommended in classical treatment is systemic antibiotic administration. Antibiotic use is also recommended metaphylactically for the protection of high‐risk animals that could foresee stress factors such as transport, weaning and dehorning (Edwards 2010). There are comparative studies on lung distributions (Foster et al. 2016) and the efficacy of several antibiotics in the treatment of BRD (Sakharkar et al. 2018). In these studies, antibiotics such as sodium ceftiofur, florfenicol, enrofloxacin, danofloxacin, gamithromycin, tildipirocin, tulathromycin and tilmicosin have been frequently studied both among themselves and with control groups to determine their use in the treatment of BRD (Ives and Richeson 2015) and for metaphylaxis (Apley 2015; DeDonder and Apley 2015).

Another pathophysiological effect of BRD is inflammation due to endotoxins, cytokines and lung parenchyma damage caused by gram‐negative bacteria that cause clinical signs such as fever, depression, anorexia and abnormal respiration in animals. Even if the disease is treated clinically, these lung lesions are later seen in slaughterhouses. Some researchers recommend using anti‐inflammatory drugs to reduce these symptoms, increase appetite and eliminate inflammation in the lung parenchyma (Bosch et al. 2004).

The primary aim of this study was to determine the treatment efficacy of nebulized florfenicol in calves naturally infected with respiratory system disease complex. Secondly, it aimed to isolate the causative agents in bronchoalveolar lavage fluid samples to determine the disease agents' distribution and follow the changes in lung ultrasonography scoring before and after treatment.

## 2 Materials and Methods

The animal material for the study consisted of 45 calves aged between 2 and 6 months, representing various breeds. These calves were presented to the animal hospital with respiratory system complaints between January 2018 and August 2019 and had not received prior antibiotic treatment or respiratory system vaccinations.

**FIGURE 1:**
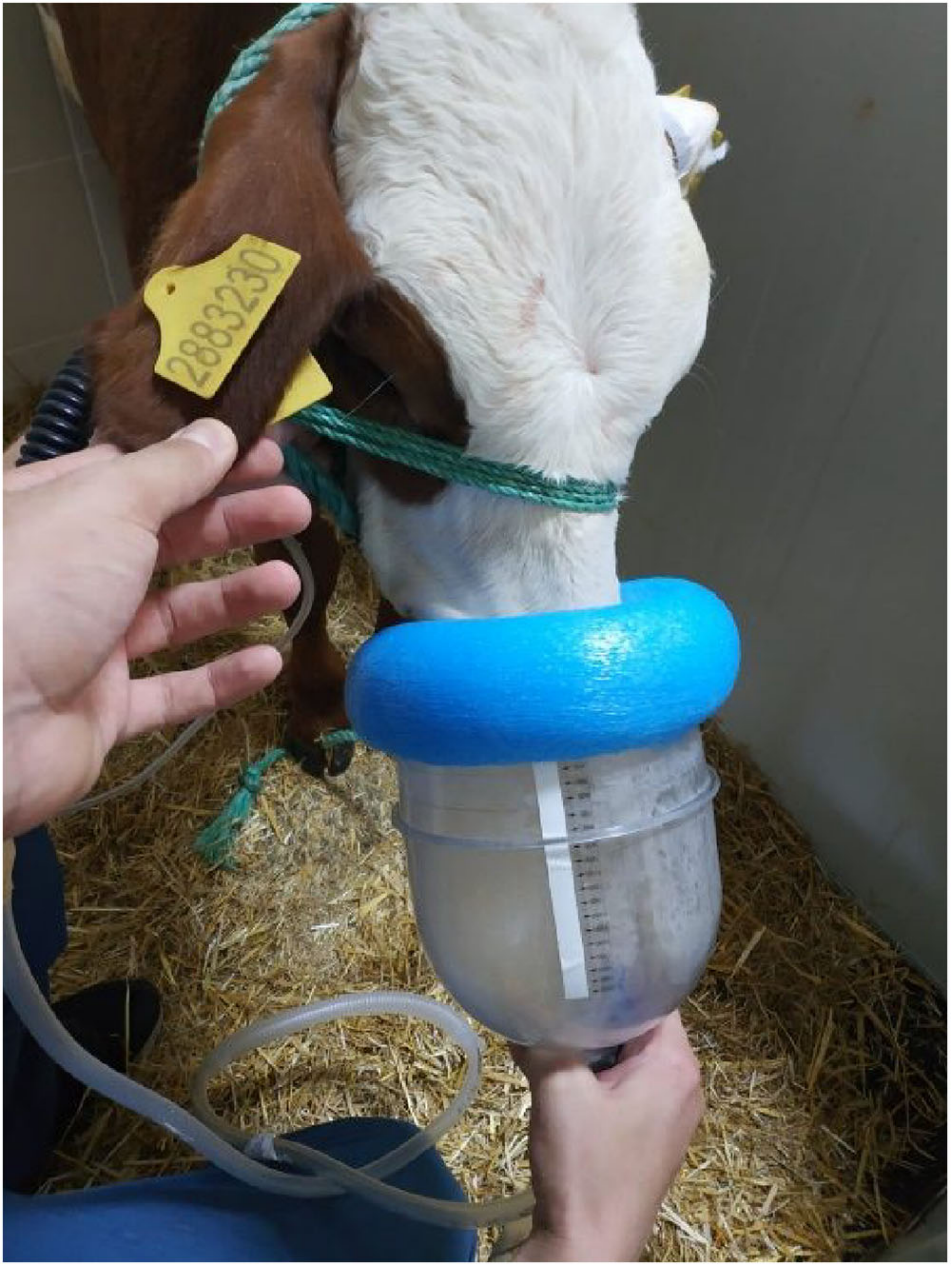
Administration of florfenicol by nebulization in a calf.

The calves were scored by the California Bovine Respiratory Disease scoring system for preweaned dairy calves (CA BRD3 scoring system) (Love et al. 2014). They were considered BRD positive if the score was higher than five. All animals were hospitalized for 7 days during the study period. The study was conducted in three groups, and BRD‐positive calves were consecutively assigned to one of these groups according to their arrival date. In Group 1, 40 mg/kg SC florfenicol (Florek, Ceva, France) and 2.2 mg/kg IM flunixin meglumine (Flumed, Alke, Türkiye) were administered once. In Group 2, 40 mg/kg florfenicol (Florek, Ceva, France) was administered via nebulization. In Group 3, nebulization of 40 mg/kg florfenicol (Florek, Ceva, France) and IM injection of 2.2 mg/kg flunixin meglumine (Flumed, Alke, Türkiye) were administered once. For the nebulization application, an Omron C28 P compressor nebulizer, which has the ability to form particle sizes of 1 to 5 mm, was used. A gas anaesthesia machine pipeline was used between the mask and the nebulizer, and the calves were nebulized with an anaesthesia mask (Figure 1). Forty mg/kg of florfenicol was added to the chamber of the nebulizer, and nebulization was continued until it was completely gone.

The calves were scored daily using the CA BRD3 scoring system during the hospitalization period. Thoracic ultrasonography was done as previously reported (Buczinski et al. 2013) and thoracic ultrasound score was noted on days 0 and 7 (Ollivett and Buczinski 2016).

**FIGURE 2:**
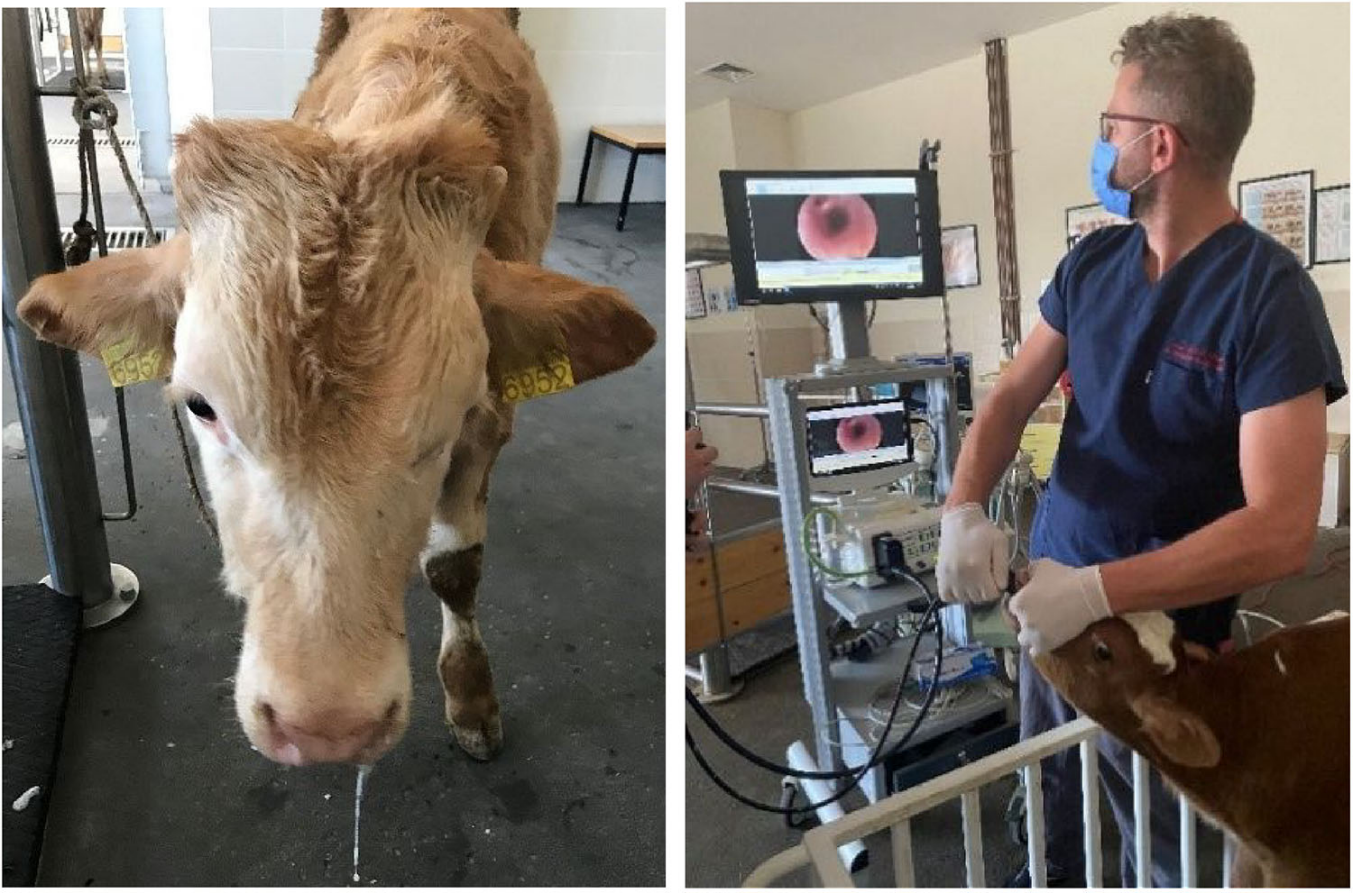
Nasal discharge in BRDc and tracheobronchial lavage sampling.

The calves were sampled for bronchoalveolar lavage fluid (Van Driessche et al. 2016) and deep nasopharyngeal swabs before being assigned to any group to determine bacterial (*P. multocida, M. haemolytica, M. bovis, Ureaplasma* spp. and *Escherichia coli*) and viral (BRSV, PI3, BHV‐1 and BVDV) agents, and samples were stored at −20°C until analysis is done. To sample BAL fluid, 0.6 mL/kg of sterile isotonic sodium chloride solution at body temperature was administered to the bifurcation site of the trachea, and at least a 5 mL sample was aspirated back (Figure 2).

Blood samples were collected at 0, 3, 5 and 7 days to perform blood counts and determine haptoglobin (Hp) levels in EDTA and plain tubes. Haematological analyses were performed immediately after sampling with Mindray BC‐5000Vet Auto analyser (China).

To investigate the viral agents (BVDV, BHV‐1, PI3 and BRSV) antigens, the multiscreen antigen ELISA Bovine Respiratory kit (Bio‐X diagnostic, Belgium, Cat Nr: BIO K340/5) was used. Results were evaluated according to the producer's criteria.

Nasopharyngeal swab samples were cultured on blood agar. DNA extractions were performed directly from both the growing bacteria in culture and bronchoalveolar lavage samples before PCR (Savaşan et al. 2016). PCR identification was modified and optimized as the protocol for *E. coli* (Abd El‐Razik et al. 2010), *M. bovis* (Akan et al. 2014), *Ureaplasma diversum* (A. Smith et al. 2012), *P. multocida* and *M. haemolytica* (Deressa et al. 2010).

### 2.1 Statistical Analysis

To estimate the required sample size, G*Power software was used to conduct a statistical power analysis with *α* = 0.05 and power = 0.80. The effect size was calculated at 0.25 (small effect size) on the data published before (Joshi et al. 2017). Under these conditions, the output was 12 calves per group, but the study was designed as 15 due to the possibility of data loss (exclusion from the study due to death).

Statistical analysis was performed with the SPSS (version 15.0 Windows) statistical analysis program. Normal distribution was assessed with the Shapiro–Wilk test. Relations (clinical respiratory score, Hp levels, haematological results) within the same groups on different days were analysed with ANOVA or the Kruskal–Wallis *H* test. Other groups on the same days were analysed with repeated measure ANOVA or Mann–Whitney *U* test. The respiratory system ultrasonographic scores before and after treatment were evaluated using the paired sample *t*‐test for inter‐group comparisons and the ANOVA method for within‐group variance analysis on the same days. The descriptive statistics were given as mean and standard deviation. A statistical significance was considered when *p* < 0.05.

## 3 Results

No side effect, such as redness, swelling or increased sensitivity at the injection site, was observed in Groups 1 and 3 during the treatment. In addition, complications such as dyspnoea and tracheal collapse did not occur in animals after nebulization.

The pre‐treatment clinical respiratory scores were 9.50 ± 1.73, 9.50 ± 1.24 and 9.33 ± 1.96 for Groups 1, 2 and 3, respectively. After 24 h of treatment protocol, clinical respiratory scores lowered in each group. In Groups 1 and 2, one calf died in the first 24 h of treatment, but not in Group 3. On the second day of treatment, the mean clinical respiratory scores of Groups 2 and 3, i.e., the groups that received nebulization, were statistically lower than Group 1, i.e., below 5, which is considered healthy. There was no significance in clinical respiratory scores between groups on the sixth and seventh days of the treatment. The daily clinical scores and calf numbers of the groups are summarized in Table 1. Three calves per group died during the study period.

**TABLE 1:** Means of daily clinical respiratory scores (mean ± SD) of the groups.

DAY	0	1	2	3	4	5	6	7
GROUP 1	9.50 ± 1.73_a_ (*n * = 15)	8.16 ± 1.80_b_ (*n * = 14)	6.83 ± 1.58_Ac_ (*n * = 13)	6.16 ± 2.32_Ad_ (*n * = 13)	4.66 ± 2.60_Ae_ (*n * = 12)	3.50 ± 2.27_Af_ (*n * = 12)	2.33 ± 1.43_f_ (*n * = 12)	2.00 ± 0.85_f_ (*n * = 12)
GROUP 2	9.50 ± 1.24_a_ (*n * = 15)	7.00 ± 2.76_b_ (*n * = 14)	4.16 ± 2.75_Bc_ (*n * = 13)	3.16 ± 1.99_Bd_ (*n * = 12)	2.50 ± 1.73_Be_ (*n * = 12)	1.66 ± 1.87_Bf_ (*n * = 12)	1.50 ± 1.93_f_ (*n * = 12)	1.16 ± 1.33_f_ (*n * = 12)
GROUP 3	9.33 ± 1.96_a_ (*n * = 15)	6.50 ± 2.43_b_ (*n * = 15)	3.83 ± 1.58_Bc_ (*n * = 15)	2.83 ± 1.33_Bd_ (*n * = 14)	2.16 ± 1.02_Be_ (*n * = 12)	1.83 ± 1.33_Be_ (*n * = 12)	1.66 ± 1.15_e_ (*n * = 12)	1.33 ± 0.98_e_ (*n * = 12)

Different lowercase letters in the same row and different uppercase letters in the same column indicate statistical significance.

Analysis of intra‐group clinical score changes revealed that Group 3 exhibited the most rapid clinical improvement.

**FIGURE 3:**
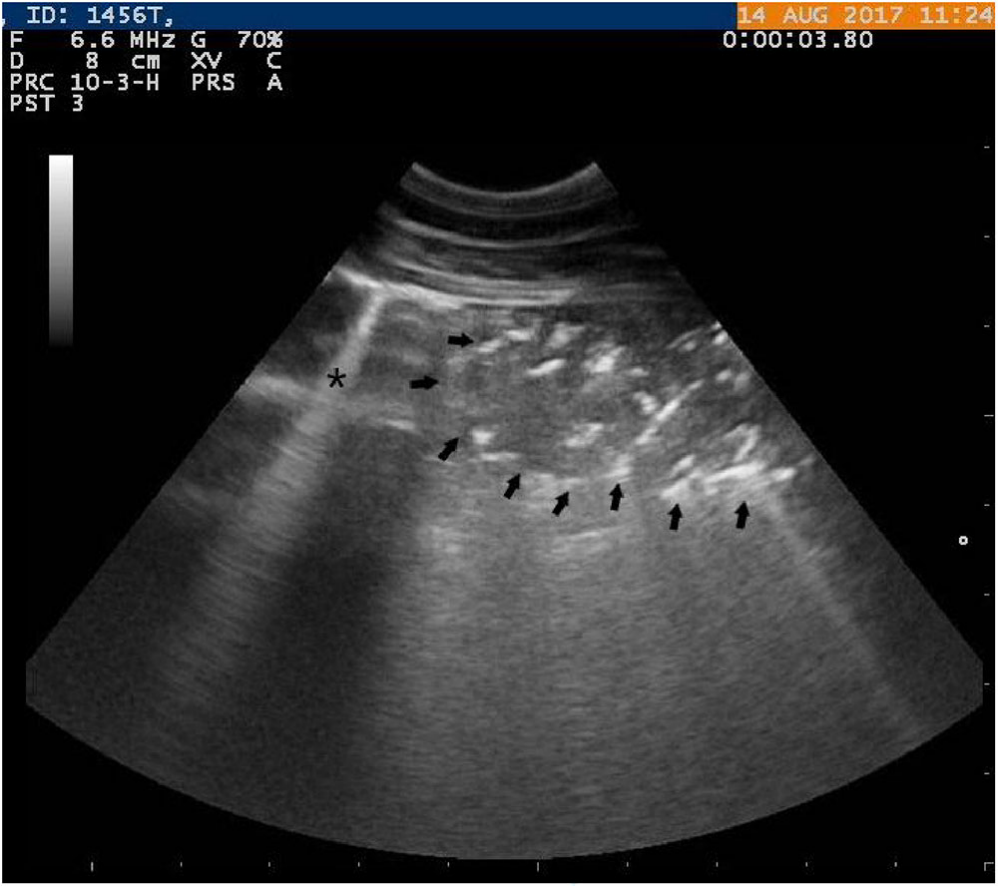
Ultrasonographic appearance of broncopneumonia (arrows) in calf. Star = comet‐tail artefact.

There was no change in the ultrasonographic score of the lung in the ultrasonographic scoring performed after treatment. Results showed that 11 of them had 5th, 3 of them had 4th and 1 of them had 3rd‐degree lung ultrasonographic scores in Group 1; 13 of them had 5th and 2 of them had 4th‐degree scores in Group 2; and all of them had 5th‐degree ultrasonographic scores in Group 3 (Figure 3).

When aetiologic agents were analysed (Table 2), *E. coli* 41 (91.1%), *M. bovis* 40 (88.8%), BRSV 21 (46.6%), *Ureaplasma* spp. (44.4%), *P. multocida* 6 (13.3%), *M. haemolytica* 4 (8.8%), BHV‐1 4 (8.8%) and BVDV 2 (4.4%) calves were positive. *Ureaplasma* spp. was isolated as the only agent in one calf. PI3 was not detected in any calf.

**TABLE 2:** Rate of mixed disease in terms of disease agents.

	Number of agents				
	1	2	3	4	5
**Group 1**	0	6 (1)	4 (2)	4	1
**Group 2**	0	4 (1)	5 (2)	5	1
**Group 3**	1	4 (1)	3	5 (1)	2 (1)
**Total**	**1**	**14**	**12**	**14**	**4**

Numbers in parentheses indicate the number of calves that died during the study period.

Although the decrease in serum Hp levels from the third day after treatment was found to be statistically significant (*p* < 0.05) in all groups, pre‐ and post‐treatment levels were found to be higher than the reference values (0–10 mg/dL) in healthy animals. Hp levels are summarized in Table 3.

**TABLE 3:** Changes in daily haptoglobin value of treatment groups.

HAPTOGLOBIN (MG/DL)	DAY 0	DAY 3	DAY 5	DAY 7
GROUP 1	95.66 ± 43.41_A_ (*n * = 15)	81.47 ± 40.92_A_ (*n * = 13)	51.70 ± 38.41_B_ (*n * = 12)	74.23 ± 38.93_C_ (*n * = 12)
GROUP 2	122.84 ± 63.73_A_ (*n * = 15)	109.29 ± 53.52_A_ (*n * = 12)	83.15 ± 29.17_B_ (*n * = 12)	71.26 ± 30.27_C_ (*n * = 12)
GROUP 3	83.95 ± 24.79_A_ (*n * = 15)	48.64 ± 28.21_A_ (*n * = 14)	48.64 ± 28.21_B_ (*n * = 12)	42.95 ± 35.35_C_ (*n * = 12)

Different letters in the same row indicate statistical difference between averages (mean ± SD).

In the pre‐treatment assessment of total leukocyte values among the groups, Group 3 showed a significant reduction compared to the other groups. After treatment, no difference was found in total leukocyte values among the groups. A significant decrease was observed in the total leukocyte value between pre‐treatment and day 3 in Group 2. Upon further examination of other haematological parameters, no statistically significant differences were observed within the groups on various days or among the same groups on the same days (Table 4). The owners were called 3 weeks after the treatment and it was learnt that there was no recurrence of pneumonia.

**TABLE 4:** Distribution of haematologic findings in treated calves according to groups.

Haematological parameters (days)	Group	Day 0	Day 3	Day 5	Day 7
WBC (×10^3^)	1	11.19 ± 5.70_aA_	15.35 ± 3.94_aA_	10.13 ± 1.41_aA_	9.96 ± 1.82_aA_
	2	14.43 ± 5.80_aA_	10.24 ± 3.56_bB_	9.50 ± 4.15_aB_	7.52 ± 3.49_aB_
	3	7.56 ± 4.67_bA_	8.84 ± 2.49_bA_	6.50 ± 1.98_bA_	8.53 ± 3.39_aA_
Neutrophile (×10^3^)	1	5.46 ± 3.31	7.35 ± 3.31	3.31 ± 3.44	4.26 ± 1.65
	2	9.73 ± 5.30	5.42 ± 4.64	4.42 ± 3.28	4.44 ± 2.33_B_
	3	4.06 ± 3.76	4.67 ± 2.60	2.35 ± 1.05	4.29 ± 3.14
Haematocrit (%)	1	26 ± 07	25 ± 04_c_	23 ± 09_c_	26 ± 07
	2	27 ± 06	32 ± 01_d_	34 ± 03_d_	32 ± 02
	3	28 ± 05	30 ± 05_c_	29 ± 05_c_	28 ± 12
RBC (×10^6^)	1	7.38 ± 2.15	6.04 ± 0.6_f_	8.05 ± 2.02	8.21 ± 2.31
	2	8.21 ± 2.08	9.65 ± 0.93_g_	10.28 ± 1.25	9.67 ± 0.93
	3	8.95 ± 3.07	9.40 ± 2.34_g_	8.16 ± 2.0	9.68 ± 2.29
Haemoglobin (g/dL)	1	8.92 ± 2.33	8.48 ± 1.17	7.90 ± 2.54	9.19 ± 2.64
	2	9.63 ± 2.20	11.06 ± 0.6	11.82 ± 1.10	11.33 ± 1.19
	3	9.64 ± 3.02	10.43 ± 2.03	10.15 ± 1.95	1.35 ± 2.29

There is a statistically significant difference between different letters in terms of averages between columns (*p* < 0.05). There is a statistically significant difference between different letters in terms of averages between rows (*p* < 0.05).

## 4 Discussion

### 4.1 Clinical Respiratory Score and Treatment Monitoring

Various diagnostic modalities, such as thoracic ultrasonography, osculation, measurement of acute phase proteins (AAPs) and haematological analysis, are utilized in diagnosing BRD. These techniques have been extensively evaluated regarding their specificity and sensitivity and are effective diagnostic tools (Buczinski et al. 2014). However, it is important to note that the cost of these methods can be substantial and their applicability may be limited in field conditions unless the disease is present as a herd problem. As such, necropsy and isolation of the causative agents remain the gold standard for the definitive diagnosis of BRD (B. P. Smith 2014).

Many researchers have developed clinical scoring systems for diagnosing BRD to facilitate rapid identification by animal owners, caregivers and veterinarians. The CA BRD3 scoring system has the advantage of requiring less manipulation of animals during scoring and of enabling accurate diagnosis with less experience through simplifying criteria (Love et al. 2014).

Additionally, the CA BRD3 scoring system accurately diagnoses BRD in 90.2% of cases (Love et al. 2014). The CA BRD3 scoring system was also used for monitoring the progression of the disease in this study. In our research, the daily variations in clinical respiratory scoring have facilitated the objective tracking of the progression.

In a study, the earliest clinical improvement was observed on the fifth day of treatment with ceftiofur administered via nebulization, which was found to shorten the therapy's duration to a conventional method (Joshi et al. 2017). The success of treatment in the group treated with nebulization was statistically significant. Clinical score was used to monitor the severity and condition of the disease in cattle and sheep treated with intramuscular amoxicillin trihydrate and reported that the clinical score reached normal limits on the fifth day (Hussein et al. 2018). It was found that the clinical score reached the level of healthy animals (< 5) on the 5th day and that the decrease on the 10th day was significant. However, the changes in the clinical scoring later on, during the 20th and 30th days, were not statistically significant. In our study, the clinical improvement of BRD‐affected calves with nebulization was faster on the second day, and thus, it was superior to the other groups.

The requirements for treatment success include respiratory score, appetite, mental state and response to treatment within 3–5 days, or observation of the type of breathing. Therefore, the treatment process is not subject to quantitative evaluation in trials. The clinical respiratory score used in this study was used for monitoring purposes, providing insight into the prognosis of the disease (DeDonder and Apley 2015). During the study, it was observed that the first‐day clinical scores of the animals that died were 12 or higher (data not shown). Based on the treatment applied, it was concluded that the prognosis of animals with clinical scores of 12 or higher was poor. Another study found that 1/6th of the animals treated with nebulization and 2/6th treated with intramuscular administration died during the study period. However, the clinical scores of the dead animals were not evaluated separately, and only group averages were given (Joshi et al. 2017).

Upon analysing the clinical scoring parameters individually, it was determined that in all groups, the initial body temperature dropped below 39.2°C (data not shown). Although the regression of clinical symptoms such as nasal discharge, eye discharge, head inclination and type of respiration occurred at different times, coughing was the last clinical symptom to disappear or even persist in animals that had recovered (those with a clinical score < 5) (data not shown). Another clinical sign observed within the 0–7 days range was that in animals whose scores dropped below 5, their appetite began during the next clinical examination. In other words, animals with a clinical score of less than 5 began to eat within 24 h. The basic pathophysiological result of BRD is the formation of clinical symptoms such as fever, depression, anorexia and abnormal respiratory functions caused by endotoxins, cytokines and inflammatory cells secreted by gram‐negative bacteria, which cause tissue damage in the lungs (Griffin et al. 2010). Therefore, even though clinical recovery occurs, the lesions that cause decreased performance in the lungs remain as a sequel and are often encountered incidentally at the abattoir (Schneider et al. 2009; Wittum et al. 1996). Therefore, anti‐inflammatory agents are believed to be necessary to alleviate the clinical symptoms of BRD (Elitok and Elitok 2004; Lockwood et al. 2003), stimulate water and feed intake (Elitok and Elitok 2004) and reduce inflammatory damage in lung parenchyma (Van De Weerdt and Lekeux 1997).

No group in the study received florfenicol via subcutaneous administration alone. The concurrent use of antibiotics and NSAIDs in conventional treatment yields superior results. In our study, we aimed to gain insights into the superiority of NSAID usage in combination with nebulization therapy. In a study, clinical recovery (fever, respiratory rate and clinical score) was significantly slower in the group that received antibiotics alone than in the group that received antibiotics along with flunixin or diclofenac (Guzel et al. 2010). In this study, clinical recovery was also observed to take shape significantly faster in the groups that received nebulized treatment than in the groups that received conventional treatment. Furthermore, the rate of recovery in the group that received nebulized florfenicol and flunixin (Group 3) was faster than that in the group that received only nebulized florfenicol (Group 2), but no statistically significant difference was found between these two groups. This finding is similar to the data of researchers Art et al. (2007), Joshi et al. (2017), and Sustronck et al. (1995), who suggest that the administration of drugs through the nebulization route is more effective. It is believed that higher population studies are needed to better evaluate the effect of non‐steroid anti‐inflammatory drugs as an ancillary treatment with nebulization therapy.

### 4.2 Ultrasonography Score

In the present study, it was revealed that although there was clinical improvement in the calves that developed BRD, lung lesions continued to persist, which highlights the importance of thoracic ultrasonography as a diagnostic tool for both early detection and monitoring of subclinical respiratory diseases in cattle.

In the diagnosis, prognosis and evaluation of treatment for respiratory system diseases, ultrasonography of the lungs is an important contributor. In recent years, many studies have been conducted on the principles of ultrasonic examination and the ultrasonic scoring of lung lesions in cattle (Fiore et al. 2022). Researchers have used a scoring system of 0–5 based on the size of the affected area to gain information on the severity of the disease (Buczinski et al. 2014; Jung and Bostedt 2004; Ollivett and Buczinski 2016). The researchers conclude that ultrasonography of the lungs has contributed to the diagnosis of bronchopneumonia and the determination of the possibility of recurrence (Timsit et al. 2019). A study that performed ultrasonic scoring of the lungs reported that in 6 animals, the lesions had regressed and tissue healing had occurred at the end of 30 days (Hussein et al. 2018). In contrast to the findings of this study, they reported that there was a significant difference between ultrasonic scores on days 0 and 5. However, they also noted that there was no correlation between clinical and ultrasonic scores, and that although the clinical score had decreased, the lesions in the lungs continued. In this study, there was no change in ultrasonic examination before and after treatment, and ultrasonic lung scores remained the same in animals showing clinical improvement.

It is believed that by a random screening of cows with thoracic ultrasound, measures can be taken early to prevent BRD, and knowledge of the future productivity of the cows participating in the screening can be obtained.

### 4.3 Aetiological Factors

In young ruminants, respiratory agents are contagious and could be fatal. In the aetiology of the disease, in addition to environmental factors, bacterial (*Pasteurella* spp. and *Mycoplasma* spp.) and viral (BRSV, BVDV and PI3) agents have essential roles. Animals that recover from the disease do not show sufficient development (Bilal 2013). *M. bovis*, which has an important role in enzootic pneumonia, was the primary pathogen in the study. Worldwide, after transportation, *M. bovis* is isolated in around 7% of calves, but within one week, it spreads to the entire herd and reaches up to 40%, and even in many studies, it infects 100% of the herd (Caswell et al. 2010). In this study, 14 (31%) calves were infected with four agents: *Ureaplasma* spp., *M. bovis*, *E. coli* and BRSV. The number of calves infected with three of the eight agents scanned was 12 (26%), and the number of calves infected with two pathogenic agents was 14 (31%). Only one calf had a single isolated BRD pathogen (*Ureaplasma* spp.). The most common agents in beef cattle are *M. haemolytica* and *M. bovis*. The results of this study are similar to previous study data (Becker et al. 2020; Catania et al. 2020; Klima et al. 2014).

In terms of viral agents, 21 (46.7%) calves were found to be infected with BRSV. In enzootic pneumonia, besides environmental factors, viral agents are known to be important in developing the disease by disrupting local defence mechanisms. At least one viral agent was isolated in 24 of the 45 animals. PI3 agent could not be isolated in any calf. Seroprevalence findings of viral agents of BRD in calves were determined as 61.9% BHV‐1, 57.1% BVDV, 64.2% BRSV and 90% PI3 in 42 samples (Roshtkhari et al. 2012). In another study, the seroprevalence of BHV‐1 was 61%, BVDV 53% and PI3 88% (Okur‐Gumusova et al. 2007). Since the seroprevalence studies were generally carried out in adult ruminants, the aetiological agents isolated from calves in this study differ.

Furthermore, the detection of the primary pathogen *M. bovis* in the majority of the cases, as well as the identification of multiple pathogens in some cases, emphasizes the complexity of enzootic pneumonia and the role of both environmental and infectious factors in its aetiology. Additionally, the high prevalence of viral pathogens, particularly BRSV, highlights the significance of viral agents in the development of respiratory disease in young cattle. These findings indicate that the distribution and virulence of pathogens may vary among regions and countries, highlighting the need for further studies to understand the epidemiology of respiratory diseases in cattle.

The formation of suppurative bronchopneumonia in BRD is positively correlated with the mixed course of the disease and the involvement of bacterial pathogens. The most commonly isolated opportunistic bacterial pathogens in the upper respiratory tract are *E. coli*, *Staphylococcus aureus* and *Proteus vulgaris* (Ouchriah et al. 2015). Although *E. coli* is not a primary cause of pneumonia, its association with the disease has been established (Contrepois et al. 1986), and it is capable of causing different types of pneumonia (DebRoy et al. 2008). In this study, *E. coli* was isolated in 91.1% of the calves. This high level of *E. coli* presence is noteworthy, has epidemiological significance and is consistent with previous studies (Algammal et al. 2020). The potential pathogenic role of these pathogens and the high frequency with which co‐infections occur make BRD a complex disease difficult to control (Szeredi et al. 2010). These findings suggest that factors such as the gathering of animals from different centres, inadequate care and feeding conditions and environmental effects such as transportation may play a role in the formation of the disease by inducing immunosuppression.

In this study, it was observed that three animals from each group died before the completion of the study. These animal's clinical examination scores on the first day were high (12 and above), suggesting that the disease was in an advanced stage. Furthermore, when analysed in terms of aetiology, *M. bovis* was isolated from seven of the nine dead calves, *Ureaplasma* spp. from five calves and BRSV from only three calves. *E. coli* was isolated from all these animals. This indicates that the hygiene and sanitation rules were not followed sufficiently in the care conditions of the calves.

### 4.4 Haptoglobin

APPs are sensitive biomarkers of inflammation and infection (Tothova et al. 2014) and are present in negligible amounts in the plasma of healthy animals (Gånheim et al. 2007; Heegaard et al. 2000; Petersen et al. 2004). Hp and serum amyloid A (SAA) are considered the dominant acute‐phase proteins in ruminants (Schrödl et al. 2016). These positive APPs have been shown to increase in the plasma of animals in response to bacterial and viral infections, surgical trauma and stress (Ceciliani et al. 2012). Higher serum APP levels in farm animals have been associated with inflammatory processes, including enteritis, endocarditis, urinary tract infections, pneumonia or peritonitis (Calves 2013; W. El‐Deeb et al. 2020).

Hp may be a useful diagnostic tool in the detection of BRD (Moisá et al. 2019) but cannot replace classical methods due to the fact that plasma concentration may increase for various reasons (W. M. El‐Deeb and Buczinski 2015). Serum Hp concentration is a valuable indicator for assessing the efficacy of antibacterial treatment, particularly in cases of bacterial pneumonia. Studies suggest that Hp is a helpful tool for monitoring the need for antimicrobial and anti‐inflammatory therapy in cases of bronchopneumonia in calves (Humblet et al. 2004; Joshi et al. 2017). However, it's important to note that Hp levels did not fully return to normal after 9 days of treatment despite clinical improvement in calves with BRD (Arslan et al. 2010). The study determined a decrease in Hp levels on the 7th day after treatment, but Hp did not decrease to reference values of 0–10 mg/dL. Considering that the half‐life of Hp is 4 days (Eckersall and Conner 1988), it is expected that Hp levels will take longer than 7 days to decrease to reference values despite treatment success.

### 4.5 Haematological Results

As previously mentioned, no gold standard exists for the antemortem diagnosis of BRD. The availability of automated blood analysers nowadays contributes to the use of haematology in diagnosing and monitoring systemic diseases (Roland et al. 2014). Haematological data showed no significant changes during the treatment period. Still, some studies have reported that platelet counts significantly increased (Cuevas‐Gómez et al. 2020), total leukocyte and haemoglobin levels increased (Šoltésová et al. 2015), and there was a decrease in RBC, haemoglobin, PCV and lymphocytes, along with a significant increase in WBC, neutrophil, monocyte and eosinophil levels (Ramadan et al. 2019). Additionally, some studies found higher neutrophil levels and lower basophil levels (Lindholm‐Perry et al. 2018).

The aetiopathogenesis of BRD is determined by multiple factors, including the immune status of the calf, its response to the disease (Bassel et al. 2020), the time of diagnosis and the duration of the illness, as well as environmental and animal diversity, which can lead to different haematological outcomes. This study concluded that the changes observed in the blood profile during the first 7 days of treatment were insignificant and that a single parameter alone is insufficient for diagnosis.

### 4.6 Nebulization Therapy

No adverse effects, such as anaphylaxis, tracheal collapse, or toxicity, were observed during or after nebulization application in the calves. In particular, in dyspnoeic animals with costo‐abdominal respiration, respiratory relief (reduction in respiratory rate and depth) was observed within a few hours after nebulization application (clinical observation).

Due to the variable drug accumulation, determining the half‐life of drugs administered through nebulization and providing a precise drug dose in treatment is challenging. Evaluating the efficacy of drugs administered via inhalation is considered more appropriate to measure the clinical effect of the drug rather than measuring the therapeutic effect or density of the drug in BAL fluid (Valachis et al. 2015). In human medicine, a pneumonia severity index such as the clinical pulmonary infection score is used to evaluate clinical improvement in respiratory system nebulization applications with uncertain results (Lu et al. 2011). It is possible to determine the effectiveness of florfenicol in calves by evaluating the severity of pneumonia clinically without a pharmacokinetic study on nebulization administration.

The primary option for the treatment of BRD is antimicrobial therapy (Hawley et al. 2016). Nebulization with medication is routinely used in cats and dogs (Ackermann et al. 2010; Rao et al. 2014). Additionally, it has been shown that antibiotics administered through nebulization in horses reach significantly higher pulmonary concentrations compared to intramuscular or intravenous routes of administration (Art et al. 2007; McKenzie and Murray 2000). Furthermore, the feasibility of administering adjunctive medications via nebulization in equine respiratory diseases has also been documented in the literature (Cha and Costa 2017).

Nebulization of amikacin in piglets has been reported to achieve lung tissue concentrations much higher than the minimal inhibitory concentration (MIC) of gram‐negative bacteria causing pneumonia (Goldstein et al. 2002). In calves with respiratory system diseases, nebulization of sodium ceftiofur has been studied experimentally (Sustronck et al. 1995) and in naturally infected cases (Joshi et al. 2017), and it is quite successful compared to intramuscular groups. Aerosol administration of drugs in cattle is limited. However, studies have shown that the applied aerosol treatment was successful compared to conventional treatment (Joshi et al. 2017). Based on the literature review, florfenicol was first used as an aerosol by nebulization in this study, and it provided earlier recovery in 2‐ to 6‐month‐old naturally infected calves with BRD.

Florfenicol is a broad‐spectrum antibiotic that exhibits bacteriostatic effects against gram‐positive and gram‐negative bacteria. Its effectiveness against *M. haemolytica*, *P. multocida* and *H. somni* has been demonstrated. In vitro susceptibility testing of *M. haemolytica*, *P. multocida* and *H. somni* isolates from cattle showed over 90% susceptibility to florfenicol (Portis et al. 2012). The concentration of florfenicol in the pulmonary epithelial surface fluid (surfactant) reached twice the therapeutic concentration, demonstrating its effectiveness in delivering the drug directly to the target tissue via nebulization while protecting against systemic side effects and achieving direct targeting of the tissue (Foster et al. 2016). The shorter duration of clinical improvement compared to conventional treatment indicates the positive effect of the method of administration, in addition to the effectiveness of florfenicol. These research findings highlight the importance of considering florfenicol's efficacy in relation to the nebulization method, which provides a means of delivering the drug directly to the target tissue and avoiding systemic side effects. The limitation of the study was that there were no negative control calves.

In conclusion, it was concluded that florfenicol administered by inhalation in BRD patients is more effective, reduces the recovery time and will be a promising treatment strategy.

## Author Contributions

**Mehmet Tutuncu**: conceptualization (equal), methodology (equal), funding acquisition, resources (lead). **Umit Ozcan**: conceptualization (equal), formal analysis (lead), methodology (equal), investigation (lead), writing–original draft preparation (lead), writing–review and editing (lead).

## Ethics Statement

Study was approved by the Ondokuz Mayıs University Ethical Review Committee (Date: 29.12.2017 Reference: 2017‐60).

## Conflicts of Interest

The authors declare no conflicts of interest.

### Peer Review

The peer review history for this article is available at https://publons.com/publon/10.1002/vms3.70238

## Data Availability

The data that support the findings of this study are openly available on ‘www.tez.yok.gov.tr‘ with thesis number 686223.
